# Racial Discrimination and Risk for Internalizing and Externalizing Symptoms Among Black Youths

**DOI:** 10.1001/jamanetworkopen.2024.16491

**Published:** 2024-06-12

**Authors:** Assaf Oshri, Ava Jane Reck, Sierra E. Carter, Lucina Q. Uddin, Charles F. Geier, Steven R. H. Beach, Gene H. Brody, Steven M. Kogan, Larry H. Sweet

**Affiliations:** 1 Department of Human Development and Family Science, University of Georgia, Athens; 2 Department of Psychology, Georgia State University, Atlanta; 3 Department of Psychiatry and Biobehavioral Sciences, University of California, Los Angeles; 4 Center of Family Research, University of Georgia, Athens; 5 Department of Psychology, University of Georgia, Athens

## Abstract

**Importance:**

Racial discrimination is a psychosocial stressor associated with youths’ risk for psychiatric symptoms. Scarce data exist on the moderating role of amygdalar activation patterns among Black youths in the US.

**Objective:**

To investigate the association between racial discrimination and risk for psychopathology moderated by neuroaffective processing.

**Design, Setting, and Participants:**

This cohort study used longitudinal self-report and functional magnetic resonance imaging (fMRI) data from Black youth participants in the US from the Adolescent Brain Cognitive Development (ABCD) study. Data were analyzed from January 2023 to May 2024.

**Exposures:**

At time 1 of the current study (12 months after baseline), youths self-reported on their experiences of interpersonal racial discrimination and their feelings of marginalization. Amygdalar response was measured during an emotionally valenced task that included blocks of faces expressing either neutral or negative emotion.

**Main Outcomes and Measures:**

At 24 and 36 months after baseline, youths reported their internalizing (anxiety and depressive symptoms) and externalizing symptoms (aggression and rule-breaking symptoms).

**Results:**

A total of 1596 youths were a mean (SD) age of 10.92 (0.63) years, and 803 were female (50.3%). Families in the study had a mean annual income range of $25 000 to $34 999. Two factors were derived from factor analysis: interpersonal racial discrimination and feelings of marginalization (FoM). Using structural equation modeling in a linear regression, standardized β coefficients were obtained. Neural response to faces expressing negative emotion within the right amygdala significantly moderated the association between FoM and changes in internalizing symptoms (β = −0.20; 95% CI, −0.32 to −0.07; *P* < .001). The response to negative facial emotion within the right amygdala significantly moderated the association between FoM and changes in externalizing symptoms (β = 0.24; 95% CI, 0.04 to 0.43; *P* = .02). Left amygdala response to negative emotion significantly moderated the association between FoM and changes in externalizing symptoms (β = −0.16; 95% CI, −0.32 to −0.01; *P* = .04).

**Conclusions and Relevance:**

In this cohort study of Black adolescents in the US, findings suggest that amygdala function in response to emotional stimuli can both protect and intensify the affective outcomes of feeling marginalized on risk for psychopathology, informing preventive interventions aimed at reducing the adverse effects of racism on internalizing and externalizing symptoms among Black youths.

## Key Points

**Question:**

How does the brain process and defend a youth from racial discrimination’s impact on mental health?

**Findings:**

This cohort study of 1596 Black youths in the US found patterns of amygdalar response that moderated the association between feelings of marginalization and changes in both internalizing and externalizing symptoms. While amygdala suppression was associated with lower externalizing symptoms, it was also associated with increased internalizing symptoms.

**Meaning:**

These findings suggest that coping with discriminatory experiences could be associated with reduced problem behaviors but increased depressive and anxiety symptoms.

## Teaser

This cohort study evaluates the association between racial discrimination and risk for psychopathology moderated by neuroaffective processing.

## Introduction

Racial discrimination refers to racially motivated discriminatory actions perpetrated by dominant racial group members toward minoritized racial group members.^1,2^ Millions of minoritized individuals^3^ are impacted by the stress and trauma associated with racial discrimination.^4^ Anti-Black discrimination in the US underlies a history of exploitation, beginning with enslavement and spanning the Jim Crow era and modern-day systematic discrimination against Black people.^5^ Among the Black youths in the US who are the focus of this study, the experience of anti-Black discrimination manifests in children’s lives through unfair treatment based on race and feelings of social-level rejection.^6,7^ Compared with their racial-ethnic minoritized peers, Black youths report the highest rates of racial discrimination in the US.^8,9^

Racial discrimination is stressful^10^ and can increase the risk for psychopathology, including both internalizing (eg, depression and anxiety) and externalizing (eg, risky behavior) problems. Differences exist in the outcomes of racial discrimination on youths’ mental health, possibly due in part to the coping strategies employed. Understanding these differences is essential for designing effective behavioral interventions alongside policy-level efforts to dismantle structural factors that underpin racial discrimination.^11,12^

Coping strategies may not be equally effective in buffering the effect of racial discrimination. Extant investigations consider the use of avoidant coping,^13,14^ yet findings are mixed. Some research suggests avoidance coping can be positive,^15^ while others have linked it to greater feelings of distress.^13,14^ Moreover, the neuroaffective processes that underlie coping with socioemotional threats may moderate the association of perceived stress and mental health risk. Understanding these mechanisms may transform the state of pediatric development science.

Neuroaffective processing linked to coping strategies may moderate the association of discrimination and mental health risk.^16^ The amygdala, crucial for emotion processing and responding to the perception of environmental threats,^17^ is critical to consider in the context of socioemotional threats such as racism.^18^ Studies on coping strategies show associations change depending on amygdalar activation during the processing of negative emotional facial expressions.^19^ Other research on the neuroaffective effects of coping strategies shows deactivation of threat perception circuits, especially the amygdala.^20^ For example, research suggests that emotional numbing is a common avoidance coping strategy following trauma.^21^ Although there is growing consensus that racism stressors are traumatic,^4^ less is known about how youths’ neuroaffective response patterns, as reflected in neural activation patterns, may alleviate the effect of racial discrimination and youths’ experience with racism on socioemotional hardship among Black youths.

The present study addresses an urgent need to inform prevention research on the socioemotional outcomes of racial discrimination among Black youths.^22,23^ The current study used a large longitudinal sample of Black youths in the US from the Adolescent Brain and Cognitive Development Study (ABCD) to (1) examine the role of racial discrimination in internalizing symptoms (IS) and externalizing symptoms (ES) and (2) test the moderating role of amygdalar response to threat on the association between racial discrimination and these symptoms. Given the inconsistent evidence on amygdalar hemispheric asymmetry in emotional processing across clinical and healthy populations,^24,25^ this study also aimed to clarify the role of amygdalar lateralization.

## Methods

This cohort study follows Strengthening the Reporting of Observational Studies in Epidemiology (STROBE) reporting guidelines. The institutional review board at each of the 21 data collection sites approved the study. Informed consent and assent were obtained from all participants. Additional details can be found in the eMethods in Supplement 1. Full sampling, recruitment, protocol, and procedures can be found online.^26^ In the current analysis, data were drawn from the second annual data collection period (12 months after baseline; mean [SD] age, 10.92 [0.63] years), the first wave at which discrimination measures were collected. Functional magnetic resonance imaging (fMRI) and self-report data were also drawn from the third and fourth annual data collection period (24 and 36 months after baseline, respectively). Caregivers self-reported their and their child’s race.

### Measures

#### Emotional N-Back Task

The emotional N-back is a commonly used fMRI paradigm to elicit implicit emotion and overt visual working memory processing.^27^ During fMRI, youths were presented with neutral or emotionally valenced pictures of faces. The present study used amygdala activation related to contrasting negative faces to the neutral faces control condition from 663 participants.

#### Amygdala Response to Negative Facial Valence

The current analysis considered amygdala responses in the right and left hemispheres when implicitly viewing negative faces compared with neutral ones. Data were acquired 24 months after baseline and had passed the ABCD Study’s quality control measures for fMRI as described in the ABCD release notes. The ABCD Data Analysis, Informatics, and Resource Center handled all fMRI quality control, data processing, and quantification of effects.^28^

#### Racial Discrimination

One year after baseline, at a mean (SD) age of 10.92 (0.63) years, youths responded to the 7-item discrimination questionnaire, which included questions from the 2006 Boston Youth Survey^29^ and the Measure of Perceived Discrimination.^8^ This assessment asked the children about their past-year experiences of being mistreated or feeling unaccepted due to race. The response scale ranged from 1 (almost never) to 5 (very often). We used these items to construct latent factors of interpersonal racial discrimination and feelings of marginalization (FoM). The items used in the interpersonal racial discrimination measure asked, “How often have [teachers/peers/other adults] treated you unfairly or negatively because of your racial/ethnic background?” An example item used in the FoM latent factor includes, “[How often do you] feel that others behave unfairly or negatively towards your racial/ethnic group?”

##### IS and ES

At 24 and 36 months after baseline, youths reported their IS and ES using the 19-item Child Behavior Checklist Brief Problem Monitor (BPM).^30^ The IS and ES subscales of the BPM are 6 items each. Youths were given a list of statements and asked if they apply to themselves. IS subscale example items include “feeling too fearful or anxious” and “unhappy, sad, or depressed,” and the externalizing subscale example items include “argues a lot” and “has temper tantrums or a hot temper.” The response scale ranged from 0 (not true) to 2 (very true or often true). Chronbach α was as follows for ES: 24 months after baseline, 0.65 and 36 months after baseline, 0.65; and for IS: 24 months post baseline, 0.72 and 36 months after baseline, 0.75. T scores used in the analysis were measured using standardized assessments calculated by the ABCD data team using age-corrected norms. A total of 627 participants had data on IS and ES.

##### Covariates

We have included covariates to our statistical models to adjust for confounding variables. These variables include age, biological sex, and income (1, <$5000; 2, $5000-$11 999; 3, $12 000-$15 999; 4, $16 000-$24 999; 5, $25 000-$34 999; 6, $35 000-$49 999; 7, $50 000-$74 999; 8, $75 000-$99 999; 9, $100 000-$199 999; 10, ≥$200 000) and were controlled in all analyses.

### Statistical Analysis

We tested hypotheses with a structural equation modeling framework implemented in Mplus version 8.0 (Muthén and Muthén). Missing data were estimated using full information maximum likelihood with robust SEs clustered by family identification number. All analyses were stratified by site identification number. More information on clustering is provided in the eMethods in Supplement 1.

Using confirmatory factor analysis, we tested a measurement model of racial discrimination with 2 latent factors, interpersonal racial discrimination and FoM. Model fit was assessed using standard indices,^31^ described in the eMethods in Supplement 1. Next, we tested the moderating role of amygdala activation to negative vs neutral faces on the association between the 2 latent variables (interpersonal racial discrimination and FoM) and IS and ES using latent interaction techniques (controlling for IS/ES from the previous year). We generated Johnson-Neyman plots and Simple Slopes and used a combined model^32^ to probe significant interaction effects (see the eMethods in Supplement 1 for more information). Significance was based on a 2-sided *P* value of less than .05, meaning there was a 5% risk for type I error. Using the Benjamini-Hochberg method, we adjusted significance levels (*P* values and confidence intervals [CI]) for multiple comparisons.^33^ Data were analyzed from January 2023 to May 2024.

## Results

A total of 1596 participants reported on racial discrimination. Families in the study had an average annual income range of $25 000 to $34 999. Youths were a mean (SD) age of 10.92 (0.63) years and 803 were female (50.3%). Participants lost to attrition did not differ on any study variables. Information on the measurement model can be found in the eResults in Supplement 1.

### Interpersonal Racial Discrimination and IS Outcome

We first tested the moderating role of right and left hemisphere amygdala response to implicit negative facial emotion on the association between interpersonal racial discrimination and FoM with IS. Interpersonal racial discrimination was not significantly associated with IS (β = 0.08; 95% CI, −0.05 to 0.20; *P* = .21; adjusted *P* = .18). Amygdalar response to negative emotion was not significantly associated with IS within either the left hemisphere (β = 0.08; 95% CI, −0.05 to 0.20; *P* = .23; adjusted *P* = .20) or the right hemisphere (β = −0.07; 95% CI, −0.04 to 0.22; *P* = .28; adjusted *P* = .24). Left hemisphere amygdala response did not significantly moderate the association between interpersonal racial discrimination and IS (β = 0.04; 95% CI, −0.15 to 0.23; *P* = .68; adjusted *P* = .63). Additionally, right hemisphere amygdala response was not significant in moderating the association between interpersonal racial discrimination and IS (β = −0.12; 95% CI, −0.25 to 0.01; *P* = .08; adjusted *P* = .07).

### Feelings of Marginalization and IS Outcome

**Figure 1.**
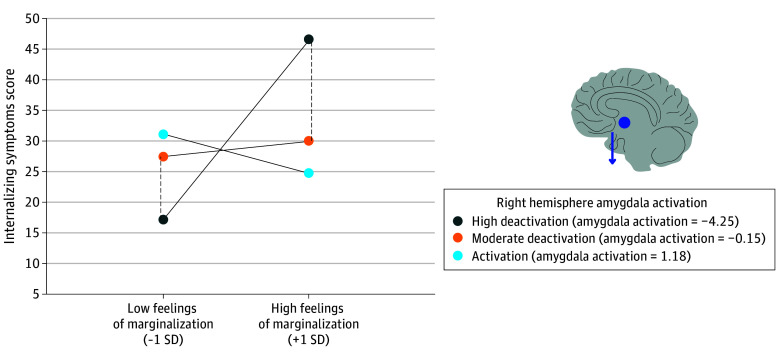
Combined Johnson-Neyman and Simple Slope Plot of the Moderating Role of Right Hemisphere Amygdala Activation on the Association Between Feelings of Marginalization and Internalizing Symptoms High and moderate deactivation are plotted at values based on the region of significance. Amygdala activation is plotted 2 standard deviations above the mean activation level. The x-axis indicates feelings of marginalization plotted 1 SD above and below the mean. The region of significance included 21% of the sample. Right hemisphere amygdala deactivation was associated with increased internalizing symptoms (depicted by dot and arrow) for youths with high feelings of marginalization.

FoM was not significantly associated with IS (β = 0.07; 95% CI, −0.04 to 0.18; *P* = .23; adjusted *P* = .20). Amygdala response to negative vs neutral faces was not significantly associated with IS in both the left (β = 0.11; 95% CI, −0.02 to 0.23; *P* = .09; adjusted *P* = .07) and the right (β = −0.09; 95% CI, −0.20 to 0.02; *P* = .11; adjusted *P* = .09) hemispheres. However, activation within the right amygdala significantly moderated the association between FoM and IS (β = −0.20; 95% CI, −0.32 to −0.07; *P* < .001; adjusted *P* = .01). The plot (Figure 1) revealed that right hemisphere amygdalar deactivation relative to negative vs neutral faces increases the association between FoM on IS. Specifically, among youths who reported high FoM, right hemisphere amygdalar deactivation response to negative vs neutral faces was significantly associated with an increase in IS. The region of significance contained 21% of the sample (335 participants). We also found that when right hemisphere amygdalar activation response to negative vs neutral faces was high, the association between FoMs on IS decreased. However, only 4% of the sample (64 participants) fell into this region of significance; thus, there was not a sufficient sample size to plot or draw conclusions.

### Interpersonal Racial Discrimination and ES Outcome

Next, we tested the moderating role of right and left hemisphere amygdala response to negative facial emotion on the association between FoMs and ES and interpersonal racial discrimination and ES in separate models. Interpersonal racial discrimination was not significantly associated with ES (β = −0.01; 95% CI, −0.12 to 0.11; *P* = .87; adjusted *P* = .83). Additionally, amygdala emotional response was not significantly associated with ES within both the left hemisphere (β = −0.03; 95% CI, −0.19 to 0.13; *P* = .72; adjusted *P* = .70) and the right hemisphere (β = −0.05; 95% CI, −0.21 to 0.10; *P* = .50; adjusted *P* = .48). Left hemisphere amygdala response was also not significant in moderating the association between interpersonal racial discrimination and ES (β = −0.01; 95% CI, −0.39 to 0.38; *P* = .97; adjusted *P* = .92). Additionally, right hemisphere amygdala response was not significant in moderating the association between interpersonal discrimination and ES (β = −0.07; 95% CI, −0.47 to 0.33; *P* = .74; adjusted *P* = .71).

### Feelings of Marginalization and ES Outcome

**Figure 2.**
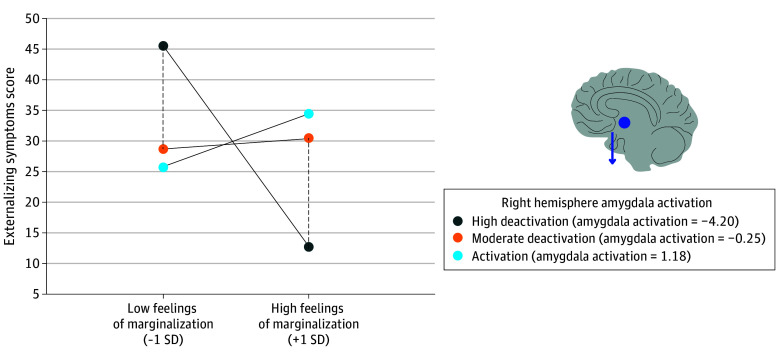
Combined Johnson-Neyman and Simple Slope Plot of the Moderating Role of Right Hemisphere Amygdala Activation on the Association Between Feelings of Marginalization and Externalizing Symptoms High and moderate deactivation are plotted at values based on the region of significance. Amygdala activation is plotted 2 standard deviations above the mean activation level. The x-axis indicates feelings of marginalization plotted 1 SD above and below the mean. The region of significance included 15% of the sample. Right hemisphere amygdala activation was associated with increased externalizing symptoms (depicted by dot and arrow) for youths with high feelings of marginalization.

FoM was not significantly associated with ES (β = −0.01; 95% CI, 0.14 to 0.12; *P* = .89; adjusted *P* = .85). Amygdala response to negative vs neutral faces was also not significantly associated with ES in both the left (β = −0.07; 95% CI, −0.15 to 0.03; *P* = .16; adjusted *P* = .15) and the right (β = 0.01; 95% CI, −0.10 to 0.13; *P* = .81; adjusted *P* = .78) hemispheres. Emotional response within the right amygdala significantly moderated the association between FoMs and changes in ES (β = 0.24; 95% CI, 0.04 to 0.43; *P* = .02; adjusted *P* = .02). The plot (Figure 2) revealed that right hemisphere amygdalar deactivation to negative vs neutral faces decreased the association between FoM on ES. Specifically, among youths who reported high FoM, low amygdala activation was protective and was associated with a decrease in ES. The region of significance contained 15% of the sample (239 participants).

**Figure 3.**
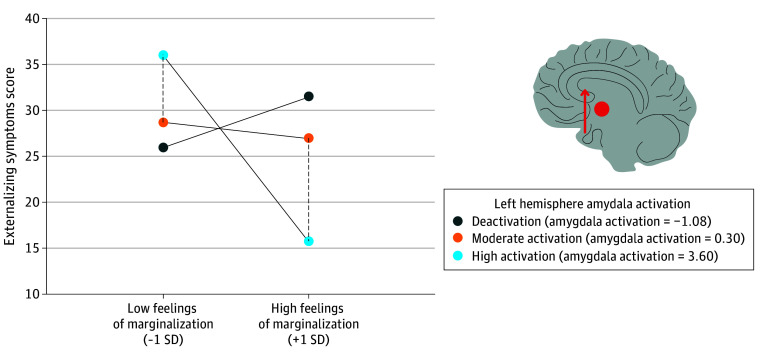
Combined Johnson-Neyman and Simple Slope Plot of the Moderating Role of Left Hemisphere Amygdala Activation on the Association Between Feelings of Marginalization and Externalizing Symptoms High and moderate activation are plotted at values based on the region of significance. Amygdala deactivation is plotted 2 standard deviations below the mean activation level. The x-axis indicates feelings of marginalization plotted 1 SD above and below the mean. The region of significance included 15% of the sample. Left hemisphere amygdala activation was associated with decreased externalizing symptoms (depicted by dot and arrow) for youths with high feelings of marginalization.

Emotional response within the left amygdala significantly moderated the association between FoM and changes in ES (β = −0.16; 95% CI, −0.32 to −0.01; *P* = .04; adjusted *P* = .03). The plot (Figure 3) revealed that left hemisphere amygdalar response to negative vs neutral faces decreased the association between FoM on change in ES. For youths who reported high FoM, a high left hemisphere amygdala response was protective and associated with decreased ES. The region of significance contained 15% of the sample (239 participants).

## Discussion

Racial discrimination experiences are stressors with serious mental health consequences for millions of Black children in the US, youths, and families in the US.^3^ Although extant research documents the outcomes of racial discrimination on risk for the development of internalizing and externalizing symptoms,^1^ less is known about the neural patterns that may protect against these experiences. In the present preliminary cohort study of Black adolescents in the US, we examined the connection between interpersonal racial discrimination and FoMs on risk for developing psychopathology (ie, IS and ES) among 1596 Black youths in the US (aged 10.92 to 14.80 years). We documented differential lateralized amygdalar activation and deactivation response patterns to negative emotional faces, showing that they moderated the association of interpersonal racial discrimination and FoMs with increased ES and IS. Given that both negative and neutral faces elicited significant activation in left and right amygdala, relative deactivation of responses during emotional face presentation compared with the neutral faces is interpreted as suppression of the amygdala during negative emotional stimulation.^34^ This interpretation is corroborated by other studies and other methods (eg, positron emission tomography) in which a decrease in amygdala activity is linked to autonomic response during noxious stimulation and self-report of coping strategies.^21^

We found that right-hemisphere amygdala deactivation response to negative emotional faces relative to neutral faces was associated with an increase in the association between FoMs on the development of IS, containing 21% of the sample in the region of statistical significance. In contrast, the deactivation pattern modulated risk for ES. The relative deactivation of the right hemisphere amygdala was associated with a decrease in the outcomes of FoMs on the development of ES. This large effect size contained 15% of the sample ( approximately 240 youths). Amygdalar deactivation may represent a neurobiological facet corresponding to emotional avoidance, which is a coping pattern to threats, such as racial discrimination. This preliminary finding may suggest that amygdalar deactivation in response to emotional stimuli is a neuroaffective survival coping response with an emotional cost. Drawing from the neuroaffective literature, suppressing emotional processing to adapt to external stress increases the risk of emotional problems.^35^

The emotional toll may reflect the physiological toll associated with racism-related stressors,^4,22,36^ while recognizing the potential adaptive necessity of this response for individuals living in an oppressive society. Research shows that many Black individuals in the US may endure under-the-skin physiological and emotional consequences, manifesting in other domains.^37^ It is plausible that Black youths in the US whose amygdala deactivates when viewing negative or threatening faces may be protecting themselves from behavioral dysregulation, such as ES, but at the cost of developing affective symptoms associated with emotional difficulties and increased IS.

The association between FoM and the development of ES over time was attenuated among youths who exhibited left-hemisphere amygdala activation. These preliminary findings validate research on amygdalar laterality in response to stress and emotional processing among human patients.^25^ Concepts related to FoMs, such as the extent to which minoritized people feel devalued or the collective perceptions and understanding of racism, may impose a psychological burden through a stressful metaperception, thereby acting as a chronic and large-scale threat.^2,38^ Because the left amygdala is implicated in detecting and responding to threats,^24^ the observed left-hemisphere activation may indicate that some Black youths in the US developed an effective way of coping with racial marginalization. Conversely, the right amygdala may be critical in detecting and responding to more immediate threatening situation,^39^ and may be linked to IS psychopathology.^40^ Thus, the observed hemispheric differences in processing racial marginalization are consistent with the hypothesis that left hemisphere deactivation can benefit behavioral regulation and reduce ES, but may incur an emotional cost.

We found no significant interaction between interpersonal racial discrimination and IS or ES, which further confirms the complexity of studying the harmful outcomes of racism and the need for studies in which mechanisms and multilevel contexts are modeled. Specifically, Black youths in the US who experience racism-related stress may be impacted very differently depending on the multiple risk and protective factors that may moderate this association. Much research has documented the harmful role of racial discrimination on adolescent well-being.^1^

Existing interventions have been shown to mitigate the outcomes of racial discrimination on mental health outcomes, such as Engaging, Managing, and Bonding through Race (EMBRace)^41^ and racism trauma-informed therapy.^42^ Interventions tailored to address racial discrimination–related stressors can potentially benefit from incorporating further understanding of the amygdala’s response to threat to develop more optimal strategies that promote adaptive emotion regulation, acknowledging the nuanced balance between behavioral control and emotional expression.

### Limitations

This study is preliminary, and further replication is required. Future studies should continue to control for the false discovery rate to reduce the risk of type I error. The extent of racism may exhibit location-based variability. We did not test the contribution of this variability in our model, but did control for demographic variables. Unique patterns of discrimination can be experienced at the individual level based on biases and prejudices against aspects of identities and social categorizations, including gender, sexual identity, class, and disability, among others.^43^ We do not focus on this issue of intersectionality in this report but recognize the critical and often multiplicative effects of bias and discrimination present in various forms. Future work must consider the complexity of discrimination to more fully address how experienced and perceived stress impacts an individual’s brain and behavior. We a priori focused on the amygdala because of its established role in threat processing.^44,45,46^ However, this does limit our study findings to the specificity of amygdala activation only. Additionally, our study did not model potential involvement of other brain regions in modulating the observed amygdalar response.

## Conclusions

This study shows that the longitudinal associations between experiences of discrimination and the risk of psychopathology among Black individuals are not necessarily direct or linear. The findings indicate that while brain deactivation patterns in the amygdala can help youths regulate problematic behaviors, this regulation comes with an emotional mental health cost. Future research should explore which regions might contribute to the amygdalar patterns identified in this study.

## Notes

Supplement 1.
eMethods.

eResults.

eReferences.

Supplement 2.
Data Sharing Statement

## References

[1] Pascoe EA, Smart Richman L. Perceived discrimination and health: a meta-analytic review. Psychol Bull. 2009;135(4):531-554. doi:10.1037/a0016059 19586161 PMC2747726

[2] Harrell SP. A multidimensional conceptualization of racism-related stress: implications for the well-being of people of color. Am J Orthopsychiatry. 2000;70(1):42-57. doi:10.1037/h0087722 10702849

[3] Lee RT, Perez AD, Boykin CM, Mendoza-Denton R. On the prevalence of racial discrimination in the United States. PLoS One. 2019;14(1):e0210698. doi:10.1371/journal.pone.0210698 30629706 PMC6328188

[4] Kirkinis K, Pieterse AL, Martin C, Agiliga A, Brownell A. Racism, racial discrimination, and trauma: a systematic review of the social science literature. Ethn Health. 2021;26(3):392-412. doi:10.1080/13557858.2018.1514453 30165756

[5] Ortega-Williams A, Harden T. Anti-Black racism and historical trauma: pushing the positive youth development paradigm. Youth Soc. 2022;54(4):662-684. doi:10.1177/0044118X211007883

[6] English D, Lambert SF, Tynes BM, Bowleg L, Zea MC, Howard LC. Daily multidimensional racial discrimination among Black U.S. American adolescents. J Appl Dev Psychol. 2020;66:101068. doi:10.1016/j.appdev.2019.101068 33994610 PMC8117402

[7] Dalal F. Racism: processes of detachment, dehumanization, and hatred. Psychoanal Q. 2006;75(1):131-161. doi:10.1002/j.2167-4086.2006.tb00035.x 16482963

[8] Nagata JM, Ganson KT, Sajjad OM, Benabou SE, Bibbins-Domingo K. Prevalence of perceived racism and discrimination among US children aged 10 and 11 years: The Adolescent Brain Cognitive Development (ABCD) Study. JAMA Pediatr. 2021;175(8):861-863. doi:10.1001/jamapediatrics.2021.1022 33999104 PMC8129899

[9] Bernard DL, Calhoun CD, Banks DE, Halliday CA, Hughes-Halbert C, Danielson CK. Making the “C-ACE” for a culturally-informed adverse childhood experiences framework to understand the pervasive mental health impact of racism on Black youth. J Child Adolesc Trauma. 2020;14(2):233-247. doi:10.1007/s40653-020-00319-9 33986909 PMC8099967

[10] Saleem FT, Anderson RE, Williams M. Addressing the “myth” of racial trauma: developmental and ecological considerations for youth of color. Clin Child Fam Psychol Rev. 2020;23(1):1-14. doi:10.1007/s10567-019-00304-1 31641920 PMC8845073

[11] Neely AN, Ivey AS, Duarte C, Poe J, Irsheid S; Transdisciplinary Resistance Collective for Research and Policy. Building the transdisciplinary resistance collective for research and policy: implications for dismantling structural racism as a determinant of health inequity. Ethn Dis. 2020;30(3):381-388. doi:10.18865/ed.30.3.381 32742140 PMC7360186

[12] Assari S. Health disparities due to diminished return among black Americans: public policy solutions. Soc Issues Policy Rev. 2018;12(1):112-145. doi:10.1111/sipr.12042

[13] Scott LD Jr, House LE. Relationship of distress and perceived control to coping with perceived racial discrimination among black youth. J Black Psychol. 2005;31(3):254-272. doi:10.1177/0095798405278494

[14] Sanchez YM, Lambert SF, Cooley-Strickland M. Adverse life events, coping and internalizing and externalizing behaviors in urban African American youth. J Child Fam Stud. 2013;22:38-47. doi:10.1007/s10826-012-9590-4

[15] Brondolo E, Brady Ver Halen N, Pencille M, Beatty D, Contrada RJ. Coping with racism: a selective review of the literature and a theoretical and methodological critique. J Behav Med. 2009;32(1):64-88. doi:10.1007/s10865-008-9193-0 19127420 PMC3258496

[16] Fani N, Carter SE, Harnett NG, Ressler KJ, Bradley B. Association of racial discrimination with neural response to threat in Black women in the US exposed to trauma. JAMA Psychiatry. 2021;78(9):1005-1012. doi:10.1001/jamapsychiatry.2021.1480 34319369 PMC8319825

[17] Whittle S, Vijayakumar N, Simmons JG, . Role of positive parenting in the association between neighborhood social disadvantage and brain development across adolescence. JAMA Psychiatry. 2017;74(8):824-832. doi:10.1001/jamapsychiatry.2017.1558 28636697 PMC5710640

[18] Liu S, Oshri A, Kogan SM, Wickrama KAS, Sweet L. Amygdalar activation as a neurobiological marker of differential sensitivity in the effects of family rearing experiences on socioemotional adjustment in youths. Biol Psychiatry Cogn Neurosci Neuroimaging. 2021;6(11):1052-1062. doi:10.1016/j.bpsc.2021.04.017 33964518 PMC8568728

[19] Drabant EM, McRae K, Manuck SB, Hariri AR, Gross JJ. Individual differences in typical reappraisal use predict amygdala and prefrontal responses. Biol Psychiatry. 2009;65(5):367-373. doi:10.1016/j.biopsych.2008.09.007 18930182 PMC2855682

[20] Allene C, Kalalou K, Durand F, Thomas F, Januel D. Acute and post-traumatic stress disorders: a biased nervous system. Rev Neurol (Paris). 2021;177(1-2):23-38. doi:10.1016/j.neurol.2020.05.010 32800536

[21] Korem N, Duek O, Ben-Zion Z, . Emotional numbing in PTSD is associated with lower amygdala reactivity to pain. Neuropsychopharmacology. 2022;47(11):1913-1921. doi:10.1038/s41386-022-01405-2 35945274 PMC9485255

[22] Brown TN, Williams DR, Jackson JS, . “Being black and feeling blue”: the mental health consequences of racial discrimination. Race Soc. 2000;2(2):117-131. doi:10.1016/S1090-9524(00)00010-3

[23] Brody GH, Chen YF, Murry VM, . Perceived discrimination and the adjustment of African American youths: a five-year longitudinal analysis with contextual moderation effects. Child Dev. 2006;77(5):1170-1189. doi:10.1111/j.1467-8624.2006.00927.x 16999791

[24] Ocklenburg S, Peterburs J, Mundorf A. Hemispheric asymmetries in the amygdala: a comparative primer. Prog Neurobiol. 2022;214:102283. doi:10.1016/j.pneurobio.2022.102283 35533810

[25] Baas D, Aleman A, Kahn RS. Lateralization of amygdala activation: a systematic review of functional neuroimaging studies. Brain Res Brain Res Rev. 2004;45(2):96-103. doi:10.1016/j.brainresrev.2004.02.004 15145620

[26] Garavan H, Bartsch H, Conway K, . Recruiting the ABCD sample: design considerations and procedures. Dev Cogn Neurosci. 2018;32:16-22. doi:10.1016/j.dcn.2018.04.004 29703560 PMC6314286

[27] Barch DM, Burgess GC, Harms MP, ; WU-Minn HCP Consortium. Function in the human connectome: task-fMRI and individual differences in behavior. Neuroimage. 2013;80:169-189. doi:10.1016/j.neuroimage.2013.05.033 23684877 PMC4011498

[28] Hagler DJ Jr, Hatton S, Cornejo MD, . Image processing and analysis methods for the Adolescent Brain Cognitive Development Study. Neuroimage. 2019;202:116091. doi:10.1016/j.neuroimage.2019.116091 31415884 PMC6981278

[29] Garnett BR, Masyn KE, Austin SB, Miller M, Williams DR, Viswanath K. The intersectionality of discrimination attributes and bullying among youth: an applied latent class analysis. J Youth Adolesc. 2014;43(8):1225-1239. doi:10.1007/s10964-013-0073-8 24318776

[30] Achenbach TM, Ivanova MY, Rescorla LA. Empirically based assessment and taxonomy of psychopathology for ages 11/2–90+ years: developmental, multi-informant, and multicultural findings. Compr Psychiatry. 2017;79:4-18. doi:10.1016/j.comppsych.2017.03.006 28356192

[31] Hu Lt, Bentler PM. Cutoff criteria for fit indexes in covariance structure analysis: conventional criteria versus new alternatives. Struct Equ Modeling. 1999;6(1):1-55. doi:10.1080/10705519909540118

[32] Roisman GI, Newman DA, Fraley RC, Haltigan JD, Groh AM, Haydon KC. Distinguishing differential susceptibility from diathesis-stress: recommendations for evaluating interaction effects. Dev Psychopathol. 2012;24(2):389-409. doi:10.1017/S0954579412000065 22559121

[33] Thissen D, Steinberg L, Kuang D. Quick and easy implementation of the Benjamini-Hochberg procedure for controlling the false positive rate in multiple comparisons. J Educ Behav Stat. 2002;27(1):77-83. doi:10.3102/10769986027001077

[34] Frank DW, Dewitt M, Hudgens-Haney M, . Emotion regulation: quantitative meta-analysis of functional activation and deactivation. Neurosci Biobehav Rev. 2014;45:202-211. doi:10.1016/j.neubiorev.2014.06.010 24984244

[35] Wilson TK, Gentzler AL. Emotion regulation and coping with racial stressors among African Americans across the lifespan. Dev Rev. 2021;61:100967. doi:10.1016/j.dr.2021.100967

[36] Bennett GG, Merritt MM, Sollers Iii JJ, . Stress, coping, and health outcomes among African-Americans: a review of the John Henryism hypothesis. Psychol Health. 2004;19(3):369-383. doi:10.1080/0887044042000193505

[37] Kahsay E, Mezuk B. The association between John Henryism and depression and suicidal ideation among African-American and Caribbean Black adolescents in the United States. J Adolesc Health. 2022;71(6):721-728. doi:10.1016/j.jadohealth.2022.07.006 36207200 PMC10405791

[38] Sellers RM, Caldwell CH, Schmeelk-Cone KH, Zimmerman MA. Racial identity, racial discrimination, perceived stress, and psychological distress among African American young adults. J Health Soc Behav. 2003;44(3):302-317. doi:10.2307/1519781 14582310

[39] Gläscher J, Adolphs R. Processing of the arousal of subliminal and supraliminal emotional stimuli by the human amygdala. J Neurosci. 2003;23(32):10274-10282. doi:10.1523/JNEUROSCI.23-32-10274.2003 14614086 PMC6741000

[40] Oshri A, Gray JC, Owens MM, . Adverse childhood experiences and amygdalar reduction: high-resolution segmentation reveals associations with subnuclei and psychiatric outcomes. Child Maltreat. 2019;24(4):400-410. doi:10.1177/1077559519839491 31030539 PMC6813855

[41] Anderson RE, McKenny M, Mitchell A, Koku L, Stevenson HC. EMBRacing racial stress and trauma: Preliminary feasibility and coping responses of a racial socialization intervention. J Black Psychol. 2018;44(1):25-46. doi:10.1177/0095798417732930

[42] Metzger IW, Anderson RE, Are F, Ritchwood T. Healing interpersonal and racial trauma: integrating racial socialization into trauma-focused cognitive behavioral therapy for African American youth. Child Maltreat. 2021;26(1):17-27. doi:10.1177/1077559520921457 32367729 PMC8807349

[43] Bauer GR, Churchill SM, Mahendran M, Walwyn C, Lizotte D, Villa-Rueda AA. Intersectionality in quantitative research: a systematic review of its emergence and applications of theory and methods. SSM Popul Health. 2021;14:100798. doi:10.1016/j.ssmph.2021.100798 33997247 PMC8095182

[44] Alexandra Kredlow M, Fenster RJ, Laurent ES, Ressler KJ, Phelps EA. Prefrontal cortex, amygdala, and threat processing: implications for PTSD. Neuropsychopharmacology. 2022;47(1):247-259. doi:10.1038/s41386-021-01155-7 34545196 PMC8617299

[45] Lindquist KA, Satpute AB, Wager TD, Weber J, Barrett LF. The brain basis of positive and negative affect: evidence from a meta-analysis of the human neuroimaging literature. Cereb Cortex. 2016;26(5):1910-1922. doi:10.1093/cercor/bhv001 25631056 PMC4830281

[46] Preckel K, Trautwein FM, Paulus FM, . Neural mechanisms of affective matching across faces and scenes. Sci Rep. 2019;9(1):1492. doi:10.1038/s41598-018-37163-9 30728379 PMC6365558

