# Supplementary material for: Racial Discrimination and Risk for Internalizing and Externalizing Symptoms Among Black Youths
Source: JAMA Netw Open. 2024 Jun 12;7(6):e2416491. doi: 10.1001/jamanetworkopen.2024.16491 (PMC11170300; doi:10.1001/jamanetworkopen.2024.16491)
Supplement: Supplement 1. — eMethods. eResults. eReferences. [file jamanetwopen-e2416491-s001.pdf]

Supplemental Online Content

Oshri A, Reck AJ, Carter SE, et al. Racial discrimination and risk for internalizing and externalizing symptoms among Black youth. *JAMA Netw Open*. 2024;7(6):e2416491. doi:10.1001/jamanetworkopen.2024.16491

eMethods.
eResults.
eReferences.

This supplemental material has been provided by the authors to give readers additional information about their work.

© 2024 Oshri A et al. *JAMA Network Open.*

eMethods

Data can be accessed through registration with the ABCD study at https://nda.nih.gov/abcd. Participants are largely recruited through the local school systems and eligibility requirements include being 9 or 10 at baseline and residing in one of the catchment areas represented by the 21 study sites[1]

Data were clustered by family and stratified by site identification in all analyses. This allows for the analysis to appropriately account for the clustering structure of families and ensure that each stratum is appropriately represented in the analysis. This method, implemented in Mplus, computes model parameters, standard errors and model fit taking into account stratification and non-independence of observations. This method is recommended for use in ABCD, and has been used in many ABCD studies[2-4]

Model fit was assed using comparative fit index (CFI), the root mean square error of approximation (RMSEA), the standardized root mean square residual (SRMR), and the chi-square statistic/degrees of freedom ratio. CFI values greater than 0.95, RMSEA and SRMR values less than 0.08, and a c2/*df* ratio less than 3.0 indicate acceptable model fit.[5]

We generated Johnson-Neyman plots and Simple Slopes and used a combined model[6] to probe significant interaction effects. The Johnson-Neyman plot is used to visually identify the direction of the association, and the regions of significance in moderation analysis. The simple slope plot depicts the slopes of the relationship between the independent and outcome variables at different levels of the moderator, aiding in interpreting how the moderator influences this relationship[7]

© 2024 Oshri A et al. *JAMA Network Open.*

eResults

The Measurement Model

The measurement model fit the data well; χ^2 (11) = 33.53, RMSEA = .04, CFI = .97, TLI = .94. All factor loadings were above 0.56, which indicates good measurement fit of factor loadings.[8]

Amygdala Activation

To assess if amygdala activation differe in response to negative versus neutral faces, we conducted t-tests comparing amygdala activation during the negative faces portion of the task to the neutral faces portion of the task. A t-test is a statistical test used to determine if there is a significant difference between the means of two groups. We found that activation during these tasks significantly differed (Left hemisphere: t[663]=2.09, p = .04, Mean difference = 0.042; Right hemisphere: t[663]=2.89, p = .00, Mean difference = 0.06), allowing us to interpret the comparison between the conditions.

Association of Covariates with Outcomes

Internalizing symptoms were not significantly associated with covariates, including sex (β= -0.27, p = 0.45), age (β = 0.01, p = 0.66), nor income (β = -0.04, p = 0.54). Additionally, externalizing symptoms were not significantly associated with covariates including sex (β = -0.05, p = 0.19), or age (β = 0.05, p = 0.17), but was significantly associated with income (β = -0.09, p = 0.04).

© 2024 Oshri A et al. *JAMA Network Open.*

eReferences

1. Garavan H, Bartsch H, Conway K, et al. Recruiting the ABCD sample: Design considerations and procedures. *Developmental cognitive neuroscience*. 2018;32:16-22.
2. Bernanke J, Luna A, Chang L, Bruno E, Dworkin J, Posner J. Structural brain measures among children with and without ADHD in the Adolescent Brain and Cognitive Development Study cohort: a cross-sectional US population-based study. *The Lancet Psychiatry*. 2022;9(3):222-231.
3. Huffman LG, Oshri A. Continuity versus change in latent profiles of emotion regulation and working memory during adolescence. *Developmental Cognitive Neuroscience*. 2022;58:101177.
4. Saragosa-Harris NM, Chaku N, MacSweeney N, et al. A practical guide for researchers and reviewers using the ABCD Study and other large longitudinal datasets. *Developmental cognitive neuroscience*. 2022;55:101115.
5. Hu Lt, Bentler PM. Cutoff criteria for fit indexes in covariance structure analysis: Conventional criteria versus new alternatives. *Structural equation modeling: a multidisciplinary journal*. 1999;6(1):1-55.
6. Roisman GI, Newman DA, Fraley RC, Haltigan JD, Groh AM, Haydon KC. Distinguishing differential susceptibility from diathesis–stress: Recommendations for evaluating interaction effects. *Development and psychopathology*. 2012;24(2):389-409.
7. Dawson JF. Moderation in management research: What, why, when, and how. *Journal of business and psychology*. 2014;29(1):1-19.
8. Comrey AL, Lee HB. *A first course in factor analysis*. Psychology press; 2013.

© 2024 Oshri A et al. *JAMA Network Open.*
