# Supplementary material for: Racial Discrimination and Risk for Internalizing and Externalizing Symptoms Among Black Youths
Source: JAMA Netw Open. 2024 Jun 12;7(6):e2416491. doi: 10.1001/jamanetworkopen.2024.16491 (PMC11170300; doi:10.1001/jamanetworkopen.2024.16491)
Supplement: Supplement 2. — Data Sharing Statement [file jamanetwopen-e2416491-s002.pdf]

Data Sharing Statement

Oshri. Racial Discrimination and Risk for Internalizing and Externalizing Symptoms Among Black Youth. *JAMA Netw Open*. Published June 12, 2024.
doi:10.1001/jamanetworkopen.2024.16491

Data
Data available: Yes
Data types: Deidentified participant data
How to access data: NDAHelp@mail.nih.gov.
When available: With publication

Supporting Documents
Document types: None

Additional Information
Who can access the data: Researchers whose proposed use of the data has been approved)
Types of analyses: Per NIH NDA data repository rules
Mechanisms of data availability: Per NDA guidelines with a signed data access agreement
Any additional restrictions: none
